# High-Temperature Sintered Conductive Silver Paste with Optimized Structure and Performance: Formula Design and Process Adjustment

**DOI:** 10.3390/nano16100606

**Published:** 2026-05-15

**Authors:** Gang Liu, Songlin Lu, Pengpeng Chen

**Affiliations:** 1 Beijing Baimtec Material Co., Ltd., Beijing 100094, China; 13651275162@163.com; 2 Key Laboratory of Materials and Application Research for Vibration and Noise Reduction, Aero Engine Corporation of China, Beijing 100097, China; 3 School of Chemistry & Chemical Engineering, Anhui University, Hefei 230601, China; sl-lu118@foxmail.com

**Keywords:** silver paste, rheological property, conductive property

## Abstract

High-temperature sintered conductive silver paste serves as a critical material in the fabrication of electronic components, with its performance directly influencing device reliability and integration density. In this work, conductive silver paste was prepared via a ball milling method by dispersing silver powder (conductive filler), glass powder (binder), and ethyl cellulose (EC, thickener) in an organic carrier composed of α-terpineol, diethylene glycol butyl ether acetate (DBA), and dimethyl phthalate (DMP) at specific ratios. The effects of the formulation composition and preparation process on the rheological properties of the paste as well as the electrical and mechanical properties of the resulting films were systematically investigated. The results indicated that sintering time and temperature exerted regular effects on the resistance of the silver paste; ball milling speed and duration influenced the particle size distribution, thereby affecting the resistance behavior; thixotropy significantly impacted the resistance characteristics. Under optimal conditions, where the organic carrier consisted of α-terpineol, DBA, and DMP at a ratio of 6:3:1, with 30 wt.% silver powder, 18 wt.% glass powder, and 4 wt.% EC, combined with a sintering temperature of 500 °C for 50–60 min, a ball milling speed of 500–600 r/min, and a ball milling time of approximately 1.5 h, the obtained silver paste exhibited pronounced shear-thinning behavior and excellent thixotropy, indicating favorable processability. The corresponding silver paste film demonstrated the lowest resistivity, superior bending resistance, and good adhesion to both PET and glass substrates. This study provides valuable insights for the design and preparation of high-performance, high-temperature sintered conductive silver pastes.

## 1. Introduction

Conductive silver paste has become an indispensable functional material in the electronics manufacturing industry due to its excellent electrical conductivity, favorable process compatibility, and cost-effectiveness [1,2,3,4]. Based on the curing process, conductive silver pastes can be classified into three categories: low-temperature curing, ultraviolet curing, and high-temperature sintering types [5,6,7]. Among these, high-temperature sintered conductive silver paste occupies a critical position in power electronic packaging, thick-film circuits, and high-temperature applications owing to its superior heat resistance, high electrical conductivity, and strong adhesion to various substrates [8,9]. However, high-temperature sintered silver pastes still face multiple challenges in practical applications: (1) conventional high-temperature sintering processes typically require temperatures ranging from 400 to 600 °C, which not only consumes significant energy but also limits their application on temperature-sensitive substrates [10,11]; (2) silver pastes are prone to pore formation and cracking during sintering, which adversely affects electrical conductivity and mechanical reliability [12]; (3) the interfacial compatibility between silver powder and glass powder [13], the volatilization behavior of the organic vehicle [14], and the rheological properties of the paste directly influence the quality and performance stability of the resulting silver film [15].

To address these challenges, researchers have conducted systematic investigations from multiple perspectives. In terms of material modification, various additives such as nano-silver particles, silver-coated copper composite powders, and diamond have been introduced to reduce sintering temperature and enhance thermal conductivity [16,17]. Regarding silver powder morphology control, different shapes (spherical, flake, dendritic) have been prepared via chemical reduction and ball milling methods to optimize particle packing density and conductive network formation [18,19]. In organic vehicle design, the volatilization and rheological behavior have been tuned by blending solvents with different boiling points and thickeners to accommodate precision forming processes such as screen printing [20].

As the core component of the conductive phase, the morphology, particle size, and distribution of silver powder play decisive roles in the electrical performance of silver pastes [21]. In recent years, extensive research has been conducted on the preparation and modification of silver powder. Through experiments, monodisperse spherical silver particles with diameters of 5–8 micrometers were successfully synthesized using a wet chemical reduction method. This demonstrated that regular morphology and uniform particle distribution facilitate the dense packing of silver particles, thereby creating more conductive pathways and enhancing electrical conductivity [22]. A systematic study was conducted examining the effect of high-energy ball milling on the microstructure of silver powder, revealing that ball milling parameters (rotational speed, duration, ball-to-powder ratio) effectively control the grain size and morphological characteristics of silver powder [23]. This demonstrates that the concentration of silver ions directly influences the rate of the reduction reaction and particle size, thereby providing theoretical guidance for size-controlled synthesis [24]. Additionally, some researchers have attempted to reduce the overall sintering temperature through surface modification or the introduction of nano-silver particles that exhibit low-temperature sintering characteristics [25]. As the binder phase, glass powder melts during high-temperature sintering to form a glass melt that wet both silver particles and the substrate surface, enhancing adhesion and promoting conductive network formation [26]. Studies indicate that the content, softening point, and wettability of glass powder significantly affect the sintering behavior and final performance of silver pastes [27]. Insufficient glass powder content leads to poor adhesion between the silver film and substrate, while excessive content hinders electron transport due to the inherent insulating nature of glass, increasing resistivity [28]. Moreover, the interfacial reaction between glass powder and silver powder is also a critical factor affecting electrical performance. However, several challenges remain to be addressed in current research. First, existing studies have predominantly focused on the independent optimization of single components (silver powder or glass powder), lacking systematic understanding of the synergistic effects among multiple components (e.g., silver powder, glass powder, and organic carrier) on overall paste performance [29]. Second, the relationship between paste rheology and printability lacks quantitative description; particularly, the mechanisms by which key rheological parameters such as thixotropy and yield stress influence fine pattern formation remain unclear [30]. Third, the effects of ball milling parameters (rotational speed, duration, media) on silver powder morphology, particle size distribution, and paste dispersibility have not been fully elucidated, with related studies remaining relatively fragmented [31].

To address the aforementioned research gaps, this work aims to systematically establish a methodology for the design and preparation of high-temperature sintered conductive silver pastes from two dimensions: formulation composition and process optimization. The main research contents include: (1) investigating the effects of key components, including silver powder, glass powder, ethyl cellulose, and the blended organic solvent (terpineol, diethylene glycol butyl ether acetate (DBA), and dimethyl phthalate (DMP)), on the rheological properties, electrical conductivity, and mechanical performance of silver pastes; (2) systematically analyzing the regulatory mechanisms of ball milling parameters (rotational speed, duration) on silver powder particle size distribution and paste dispersibility; (3) studying the effects of sintering temperature and duration on the microstructure and resistivity of silver films, revealing the microstructural evolution during sintering. Based on the optimal formulation and process window, this work provides systematic theoretical guidance for the development and application of high-performance conductive silver pastes.

## 2. Materials and Methods

### 2.1. Materials

The silver powder (micron flakes) was supplied by Shanghai Xinzuan Alloy Materials Co., Ltd., while the glass powder (industrial grade) was supplied by Xincheng Mining Products. The terpineol (C_10_H_18_O, purity: ≥98.0%) was supplied by Hefei Chengshang Laboratory Supplies Co., Ltd., Both diethylene glycol butyl ether acetate (DBA, AR) and dimethyl phthalate (DMP, AR) were procured from Sinopharm Chemical Reagent Co., Ltd. Ethyl cellulose (EC, AR) was supplied by Shanghai Titan Technology Co., Ltd.

### 2.2. Preparation of Conductive Silver Paste

The silver powder, glass powder, compound organic solvent (composed of DBA, DMP, and terpineol mixed in a specific ratio), and EC were added into a ball mill jar according to the designated proportions. Conductive silver paste with suitable fluidity was prepared by optimizing the ball milling time and rotational speed.

### 2.3. Preparation of Conductive Silver Paste Film

The paste was coated onto a polyethylene terephthalate (PET) substrate using screen printing technology. Subsequently, high-performance conductive silver paste films were obtained by precisely controlling the sintering temperature and duration.

### 2.4. Characterization

An advanced rheometer (MCR702, Anton Paar GmbH Austria) was used to test the viscosity, thixotropy, and viscoelastic behavior of conductive silver paste. The viscosity test was performed at ambient temperature with the shear rate from 1 s^−1^ to 100 s^−1^. The thixotropy of conductive silver paste was obtained by a three-stage thixotropy test (3ITT) at ambient temperature: the initial test segment at a shear rate of 1 s^−1^ for a duration of 60 s; the second test segment at a shear rate of 100 s^−1^ for a duration of 5 s; the subsequent test segment at a shear rate of 1 s^−1^ for 120 s. The viscoelastic behavior of conductive silver paste was evaluated at ambient temperature with the shear strain value from 0.1% to 100% at a constant angular frequency value of 10 s^−1^.

The conductive silver paste coated on the PET substrate was sintered, and then gold was sprayed on its surface. The surface microstructure was observed by using a scanning electron microscope (SEM; S-4800, Hitachi, Japan) A small amount of conductive silver paste was placed into a centrifuge tube, diluted with ethanol until the solution became transparent, and the particle size of the filler was then measured using a visual particle size analyzer (ViewSizer 3000, Manta Instruments Inc., USA). A small amount of the cured silver paste was placed in a centrifuge tube and dispersed in ethanol by ultrasonication for 20 min. A drop of the dispersed solution was carefully transferred onto a copper grid using a pipette. After drying, the microstructure was observed using transmission electron microscopy (TEM; FEI Tecnai G2 F20, The Netherlands). The electrical resistivity and sheet resistance were measured using the four-point probe method, in which four linearly aligned probes were vertically pressed onto the sample surface with a constant pressure. The adhesion of the conductive silver paste to the substrate was evaluated according to GB/T 9286-2021 [32]. A cross-cut pattern was created using a cross-cut tester, and a 3M tape was applied onto the cured film. After standing for approximately one minute, the tape was rapidly peeled off perpendicularly to the substrate, and the adhesion condition was observed. The silver paste film was cut into 1.5 cm × 1.5 cm pieces, and its dielectric properties were measured using a broadband dielectric impedance spectrometer (Concept40, Novocontrol GmbH, Germany) at room temperature over a frequency range of 10^−1^ to 10^7^ Hz. The experiment was carried out three times, and the results were plotted using the average value. The thermal stability and mass loss of the sample were evaluated using a thermogravimetric analyzer coupled with a differential scanning calorimeter (TG-DSC; STA-200, Shanghai Instrument Equipment Co., Ltd., China). The measurement was conducted in air atmosphere at a heating rate of 10 °C/min over the temperature range of 25–1000 °C.

## 3. Results and Discussion

### 3.1. Effect of Formula Composition on the Properties of Silver Paste

The organic carrier must have a specific evaporation rate to ensure the levelling and storage stability of the silver paste at different stages. If the organic solvent evaporates too quickly, the organic carrier and silver paste cannot be stored for long periods. Conversely, a low evaporation rate of the organic solvent hinders the drying of the silver paste and the achievement of fine patterns. It is imperative to select a blended solvent system that exhibits low volatility at room temperature and high volatility at elevated temperatures for the organic carrier [33]. The solvents selected for this study were terpineol, DBA, and DMP, with boiling points of 217 °C, 246 °C, and 282 °C, respectively. Solvents with differing boiling points were selected to optimally regulate the evaporation rate of the organic carrier. Based on the results of the system exploration (see Figures S1–S3), the ratio of terpineol, DBA, and DMP was determined to be 6:3:1.

**Figure 1:**
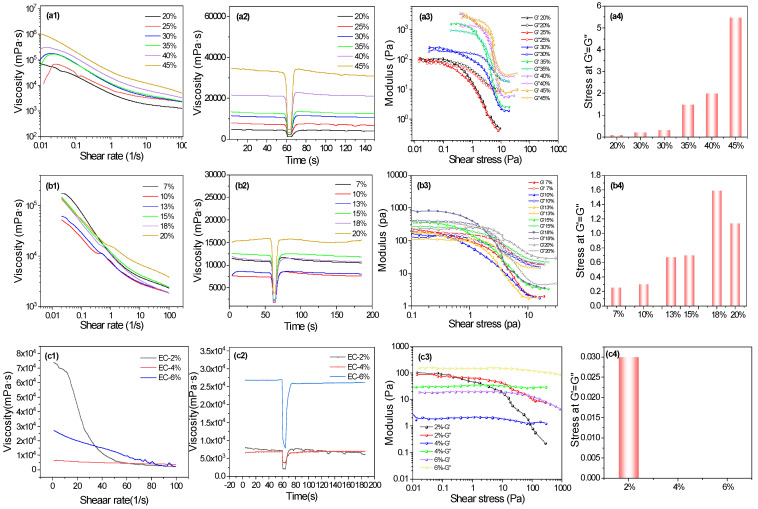
Viscosity, thixotropy, stress–strain amplitude curves, and the stress at the flow point (G′ = G″) of silver pastes containing different contents of (**a1**–**a4**) silver powder, (**b1**–**b4**) glass powder, and (**c1**–**c4**) ethyl cellulose, respectively.

The silver powder content exerts a substantial influence on the physicochemical properties of silver paste. As demonstrated in Figure 1(a1), at identical shear rates, conductive silver paste with a higher content of silver powders demonstrates greater viscosity and weaker shear-thinning behavior; at shear rates ranging from 0.01 to 100 S^−1^, the viscosity of conductive silver paste with 20–35 wt.% silver powders shows a trend of first increasing and then decreasing; however, this trend gradually dissipates as the silver powder content continues to rise. It is hypothesized that this phenomenon is due to the fact that the silver paste is more sensitive to temperature at a low shear rate, and as the content of silver powders increases, the viscosity–temperature characteristics are enhanced [34]. At elevated levels of shear rate, the silver paste’s viscosity undergoes a reduction in accordance with the magnitude of the applied shear rate, indicating the shear thinning behavior. Figure 1(a2) displays the thixotropy of the silver paste. A spontaneous rise in shear rate results in a decrease in viscosity, thereby corresponding to the pseudoplastic behavior that is evident in Figure 1(a1). It is apparent that an increase in the amount of silver powders has a negligible effect on thixotropy. However, it is notable that when the silver powder content reaches 45 wt.%, recovery occurs almost instantaneously.

The results of stress–strain amplitude testing for silver pastes containing different silver powder contents are shown in Figure 1(a3,a4). The response to applied shear stress was evaluated based on the storage modulus (G′) and loss modulus (G″), with a constant frequency of 1 Hz and an amplitude range from 0.01% to 100%. Despite the variation in G′ values, all silver pastes demonstrate an analogous dynamic behavior. Prior to the flow point (G′ = G″), the behavior of the paste is dominated by viscoelastic phenomena, with no significant structural changes occurring internally, though local irreversible deformation might occur. Beyond the flow point, where G″ exceeds G′, the paste transitions from a solid to a liquid state. Concurrently, as the viscosity of the paste progressively decreases, its internal structure gradually deteriorates. From Figure 1(a3), the modulus increases in a progressive manner as the silver powder content rises. From Figure 1(a4), the shear stress at the flow point increases in a stepwise manner with rising silver powder content, signifying the necessity for elevated levels of shear stress input. However, when silver powder content reaches 45 wt.%, the plateau phase of the modulus is virtually eliminated. It has been demonstrated that this property impedes the flow of conductive paste through the screen onto the fabric substrate during the process of screen printing. This, in turn, results in a subsequent hindrance to the release of the paste from the screen [35].

To enhance adhesion and bond strength, ensure a firm bond between the silver layer and the substrate, form a stable conductive network, and promote electrical conductivity, we need to add glass powder to achieve excellent adhesion and electrical conductivity. The effect of glass powder content on the rheological properties of the silver paste was further examined. As demonstrated in Figure 1(b1), the viscosity of silver pastes undergoes a gradual decrease in conjunction with an increase in shear rate, thereby manifesting shear-thinning behavior. From Figure 1(b2), the recovery rate of the paste initially decreases and then increases with rising glass powder content, indicating a gradual increase in the paste’s thixotropy. In the case of the silver paste with 20 wt.% glass powders, no thixotropy is observed. It is evident that, within a certain range, glass powder has the capacity to modify the paste’s thixotropic recovery behavior. As shown in Figure 1(b3), the paste continues to demonstrate a flow point as the glass powder content increases, indicating a transition from a solid to a liquid state. The modulus peak of the silver paste containing 18 wt.% glass powders is obtained. The shear stress required at the flow point increases progressively (see Figure 1(b4)), indicating a strengthening of the network structure formed within the paste. This strengthening reaches its maximum at 18 wt.% glass powder content. Experiments have shown that 18 wt.% glass powder is the optimal formulation.

The effect of EC content on rheological properties of the silver paste was also investigated. The primary function of EC as an adhesive thickener is to increase the viscosity of silver paste. This process imparts favorable rheological properties to the silver paste, thereby facilitating its application and coating. Figure 1(c1,c2) presents viscosity and thixotropy curves for silver pastes at varying EC concentrations, primarily set at 2 wt.%, 4 wt.%, 6 wt.%, 8 wt.%, and 10 wt.%. However, at levels of 8 wt.% and 10 wt.% EC content, the negligible fluidity, approaching a solid state, is observed for silver pastes. Consequently, these two EC addition levels are deemed to be unsuitable. It is evident that EC functions as a viscous thickener, with the primary function of this agent being to enhance the rheological properties of silver paste by increasing viscosity [36]. As displayed in Figure 1(c2), at a concentration of 6 wt.% EC, whilst there is considerable variation in the viscosity of silver paste, its thixotropy is largely absent. Figure 1(c3) presents stress–strain amplitude curves of silver paste with varying EC concentrations. It is evident that as the content of EC increases, both G′ and G″ values of the silver paste transition from intersecting to gradually parallel lines, with the storage modulus consistently lower than the loss modulus. This phenomenon may be attributed to the higher EC content, which appears to influence the material’s brittleness and fracture toughness, thereby increasing its susceptibility to fracture and reducing the storage modulus [37]. Alternatively, the presence of voids, bubbles, or other defects within the silver paste could increase internal friction and energy dissipation, consequently contributing to a reduction in the storage modulus [38]. Taking all factors into account and based on experimental comparisons, we have determined that the EC content in the slurry is 4 wt.%.

### 3.2. Effect of Process Parameters on the Properties of Silver Paste

Ball milling and sintering processes are crucial for achieving uniform dispersion of fillers in silver paste and ensuring the densification of the resulting film. Therefore, a silver paste with a fixed formulation (terpineol:DBA:DMP mass ratio of 6:3:1, 30 wt.% silver powder, 18 wt.% glass powder, and 4 wt.% EC) was employed to investigate the effects of processing parameters on its performance.

**Figure 2:**
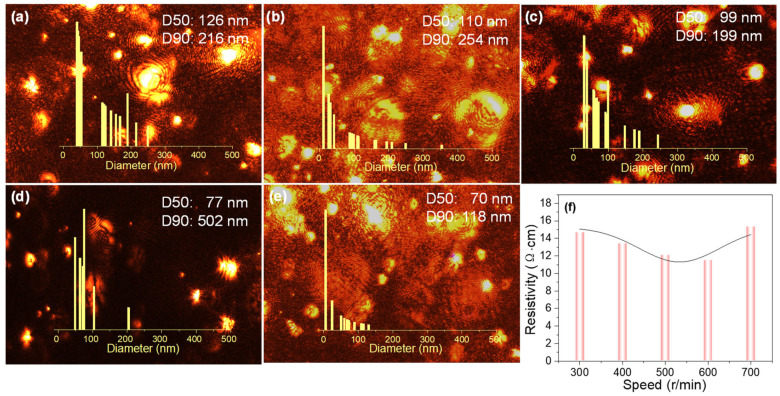
Microscopic images and particle size distribution curves for particles tested at the ball milling speed of (**a**) 300 r/min, (**b**) 400 r/min, (**c**) 500 r/min, (**d**) 600 r/min, and (**e**) 700 r/min; (**f**) Schematic of resistivity versus ball milling speed.

Figure 2 shows the particle size distribution and resistivity of silver pastes performed at various rotational speeds. As rotational speed increases, the particle size distribution shifts. In Figure 2a, approximately half the particles (D50) cluster near 126 nm, while approximately 90% of particles (D90) cluster near 216 nm. In Figure 2d,e, the D50 values are between 70 nm and 77 nm. This indicates a narrowing of the particle size distribution range, which correlates with the observed reduction in the dimensions of the bright spots in the micrograph. Clearly, the increased rotational speed leads to smaller particle sizes and promotes uniformity of size. Furthermore, the distribution exhibits a decreasing trend across different rotational speeds. Figure 2f shows the resistivity plot for the corresponding rotational speeds. It is evident that resistivity initially decreases, then increases, with increasing rotational speed. This aligns with the experimental observation that higher rotational speeds result in increased granularity on the surface of the cured silver paste film. It is hypothesized that, at higher ball milling speeds, the smaller particle size creates larger inter-particle gaps during curing, thereby reducing electrical conductivity [39].

**Figure 3:**
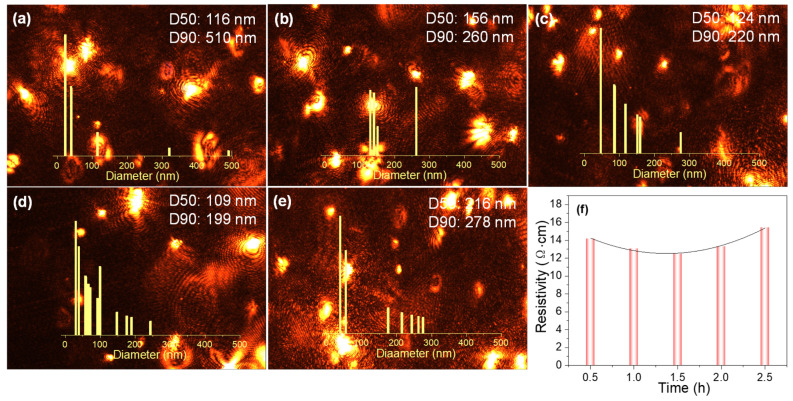
Microscopic images and particle size distribution curves for particles tested at ball milling durations of (**a**) 0.5 h, (**b**) 1 h, (**c**) 1.5 h, (**d**) 2 h, and (**e**) 2.5 h; (**f**) Schematic of electrical resistivity versus ball milling duration.

Following an investigation into the effects of ball milling speed, the subsequent examination focused on the impact of varying ball milling duration. In instances where milling time was limited, the silver paste underwent inadequate grinding, leading to a broad particle size distribution with inconsistent particle dimensions. As the milling time was progressively extended, the silver paste underwent sufficient collision and friction, gradually narrowing the gap between particle sizes. As seen in Figure 3a, the D50 and D90 value of particles is 116 nm and 510 nm, respectively; but in Figure 3e, the D90 value is around 278 nm. As demonstrated by the corresponding micrographs obtained from particle size analyses of silver paste following grinding durations of varying lengths, a gradual reduction in the number of large fluorescent bright spots becomes evident as the grinding period is extended. As illustrated in Figure 3f, the resistivity profiles of silver paste films produced under varying ball milling durations demonstrate the presence of minimum values. The findings demonstrate that both ball milling speed and duration exhibit optimal values for influencing the properties of silver pastes.

**Figure 4:**
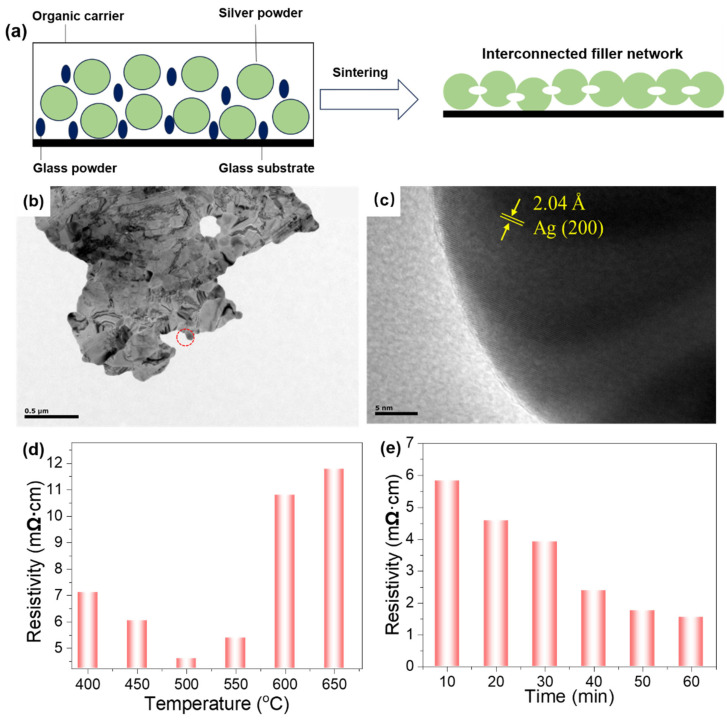
(**a**) Schematic representation of conductive silver paste before and after sintering; (**b**) TEM image and (**c**) its local magnification of the silver paste film after sintering; resistivity of silver paste films at different (**d**) sintering temperatures and (**e**) sintering durations.

High-temperature sintering of silver paste has been demonstrated to facilitate a more profound comprehension of its physicochemical properties [40], as shown in Figure 4a. As PET substrates are sensitive to high temperatures, we opted for glass substrates for high-temperature sintering. During this process, the removal of the solvent enabled the agglomeration of the silver powder into a dense structure. Figure 4b shows the dense structure formed by the plate-like silver particles after sintering. Subsequent to local magnification and data processing in Figure 4c, the lattice spacing of 2.04 Å is shown, corresponding to the (200) crystal plane of silver. This phenomenon is attributed to the ordered arrangement of silver particles during sintering, where interparticle interactions lead to the formation of lattice fringes. This finding also suggests that crystallization occurs during the sintering process, resulting in a directionally aligned lattice structure.

To evaluate the sintering temperature of silver pastes, TG-DSC analysis was performed, and the result is shown in Figure S4. Upon reaching 400 °C, the TG curve stabilizes at around 50 wt.%. By this point, the organic carrier and additives in the silver paste have been fully evaporated, leaving a residue comprising a 50 wt.% conductive phase and binder phase. The DSC curve also reveals a distinct exothermic peak near 300 °C. Given that the flash point of ethyl cellulose is 319.5 °C, this peak is likely to be due to its combustion. Additionally, the endothermic peak observed near 960 °C corresponds to the melting of the silver powder. It is considered that, during the pre-sintering heating process, the paste should be held at temperatures 5–10 °C below the boiling point of each organic carrier component for a period to ensure the carrier components evaporate completely. This facilitates subsequent sintering. Therefore, the sintering temperature for the silver paste was fixed at 400 °C.

**Figure 5:**
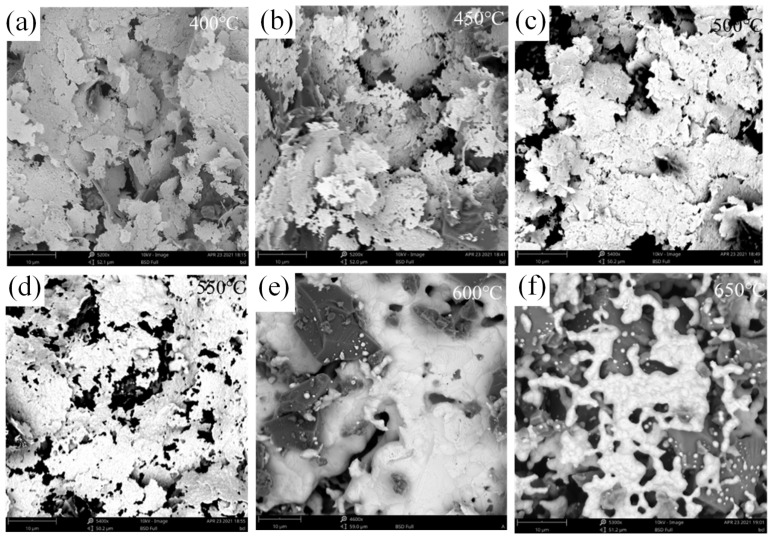
SEM images of silver paste film at different sintering temperatures: (**a**) 400 °C, (**b**) 450 °C, (**c**) 500 °C, (**d**) 550 °C, (**e**) 600 °C, and (**f**) 650 °C.

The resistivity of the sintered silver paste film was measured at 50 °C intervals throughout the process from 400 °C to 650 °C (Figure 4d). As the temperature increases from 400 °C to 500 °C, a gradual decrease in resistivity is observed. Between 500 °C and 550 °C, the resistivity exhibits negligible change. However, a significant increase in resistivity is observed between 550 °C and 600 °C. Subsequent heating results in a gradual increase in resistivity. This finding indicates that the silver paste undergoes three distinct stages during the temperature-dependent sintering process. In order to further elucidate the cause of resistivity variation, an SEM analysis was conducted, and the results are presented in Figure 5. In the initial stage, at temperatures of approximately 400 °C, the formation of flake-like, micron-sized silver powder commences, undergoing a process of sintering. At this juncture, due to the high surface energy, partial flake stacking occurs. In the second stage, as the temperature increases gradually, silver particles become more active and begin to separate from one another. The process of flake stacking decreases, and the pore depths become shallower. Upon reaching the third stage, the elevated temperatures and the binding action of the glass powder result in the mutual contact of the silver particles, causing them to fuse together. As the glass powder melts further, the silver particle concentration within the melt exceeds the saturation solubility at the surfaces of larger silver particles or in depressions within the powder. At these locations, silver precipitates and crystallizes. Furthermore, the presence of unstable grain evolution and dispersion in the particle size distribution has been demonstrated to result in increased resistivity [41].

**Figure 6:**
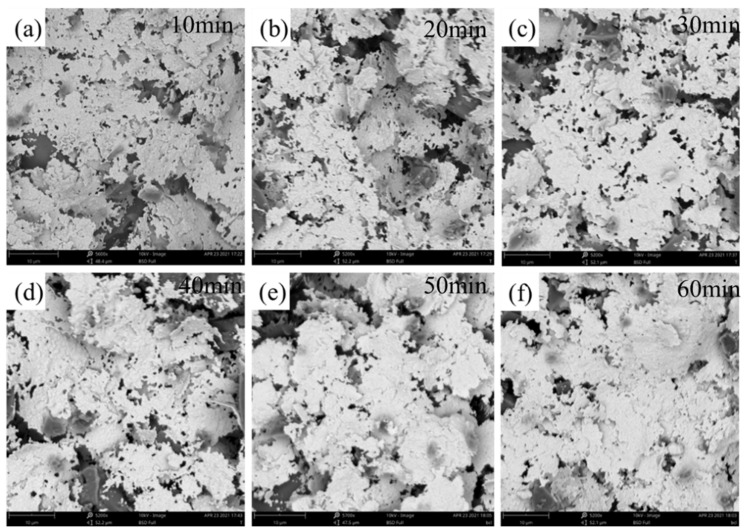
SEM images of silver paste films at different sintering times: (**a**) 10 min, (**b**) 20 min, (**c**) 30 min C, (**d**) 40 min, (**e**) 50 min, and (**f**) 60 min.

The analysis of the resistivity of the silver film at varying sintering temperatures indicates that the lowest resistivity is observed at 500 °C. The sintering temperature was set at 500 °C in order to investigate the effect of varying sintering durations on the silver paste film, with specific reference to changes in its resistivity (Figure 4e). SEM images in Figure 6 reveal that after 10 min of sintering, flake silver powders exhibit numerous cracks, with significant gaps forming between individual flakes. As sintering time is extended, the degree of densification increases gradually. The connections between the flake-like silver particles become more robust, and the large voids begin to decrease in size. As the sintering time increases beyond 50 min, achieving a duration of 10 min for the sintered sample, a significant enhancement in the degree of densification is observed. A greater proportion of flakes are found to be interconnected by glass powder, leading to a decline in the accumulation of particles. This phenomenon results in a silver paste film surface that exhibits an enhanced uniformity [42].

### 3.3. Electrical Properties of Silver Paste Films

**Figure 7:**
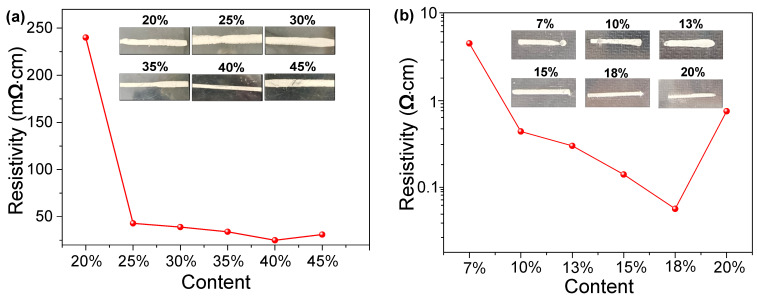
Resistivity of silver paste films with varying amounts of (**a**) silver powders and (**b**) glass powders and their screen-printing images.

The conductivity of silver paste is determined by the amount of silver powders. The experiment was carried out three times, and the results were plotted using the average value. Figure 7a shows the resistivity of silver pastes containing different silver powder content after 20 min of sintering. At a 20 wt.% silver powders, the resistivity is 238.6 mΩ·cm. As the silver content increases to 25 wt.%, the resistivity of the paste decreases rapidly. Beyond 25 wt.% silver powders, the decrease rate of resistivity becomes less pronounced. When the content reaches 40%, the resistivity minimizes to 1.08 mΩ·cm. However, when the silver powder content reaches 45 wt.%, the resistivity of silver paste film begins to show an increasing trend. As seen from the macro-printing images in Figure 7a, despite using identical screen specifications (3 cm × 1 mm mesh size) for screen printing, the printed patterns differ significantly. This correlates with the paste’s rheological properties. In previous 3ITT tests, when the silver content exceeds 35 wt.%, a delayed viscosity recovery is observed. The pastes with silver content ranging from 20% to 30% exhibit uneven printed lines post-printing. This is due to inadequate thixotropy, signifying that the silver paste’s viscosity recuperates too expeditiously, which has an unfavorable effect on print quality. It can be established that the printed lines exhibit optimal uniformity when the silver content is set at 40 wt.%.

To further analyze the effect of the content of silver powder on the electrical properties of silver paste, the surfaces of silver paste films prepared with varying contents of silver powder were observed by SEM (Figure S5). As the temperature increases, the organic carrier begins to volatilize. Once fully volatilized, the organic carrier causes the glass powder to soften into a glass melt. Due to the excellent wetting effect of the glass powder on the silver particles, surface contact between the silver powder and the substrate is enhanced. This promotes the formation of a dense conductive network between the paste and the substrate, thereby improving the paste’s adaptability to different applications [26]. Figure S5a illustrates that the lowest silver content results in poor connectivity between silver sheets post-sintering. At 40 wt.% silver content (Figure S5e), a dense interconnection becomes apparent. However, further increasing the silver content introduces larger voids (Figure S5f), consequently leading to an increase in resistivity.

The influence of glass powder content on the resistivity of silver paste films was also examined. The experiment was carried out three times, and the results were plotted using the average value. The test results are presented in Figure 7b. Resistivity is seen to initially decrease, then increase with rising glass powder content. The resistivity starts at 3.83 Ω·cm with 7 wt.% glass powder, decreases to 0.037 Ω·cm with 18 wt.% glass powder, and then rises again to 0.54 Ω·cm at 20 wt.% glass powder. Increasing the mass fraction of glass powder in the silver paste enhances adhesion between the film and substrate, establishing favorable contact conditions for ohmic bonding. However, the inherently poor conductivity of the glass melt itself becomes problematic at higher concentrations. This impedes electrical conduction between the film and substrate, consequently elevating the contact resistivity [28]. Screen printing tests were conducted on silver pastes with varying glass powder contents. Referring to the previous renogram of the glass powder (Figure 1(b2), at a glass powder mass fraction of 7%, the thixotropic recovery is too rapid and the required shear stress is too low. This results in the aforementioned breakpoint. At 10 wt.% and 13 wt.% glass powders, some thixotropic recovery is present, but the viscosity is too low. This causes the paste to bleed outwards during screen printing, resulting in increased width. As the glass powder content increases further, the silver paste exhibits improved thixotropic recovery and higher shear stress, resulting in more uniform screen-printed patterns. However, the increased glass powder content also impedes the electron transport.

After determining the resistivity of silver paste films containing different concentrations of glass powder, SEM analysis was conducted to investigate the microstructure of each sample. The results are presented in Figure S6. It can clearly be seen that, at elevated temperatures, the glass powder begins to melt, which enhances the bonding effect for the silver powder. As the glass powder content increases progressively to 18 wt.% (Figure S6e), the sample’s morphology evolves from initially discontinuous and porous connections to dense, plate-like connections forming. This enhanced densification improves conductive performance, thereby reducing resistivity [43]. However, further increasing the glass powder content, as shown in Figure S5f, results in the accumulation of flake silver and large glass powder particles, which obstruct the formation of conductive pathways. This aligns with the aforementioned changes in resistivity. Therefore, following high-temperature sintering, the silver paste operates primarily via a contact-based conductivity mechanism.

### 3.4. Physicochemical Properties of Conductive Silver Paste Films

**Figure 8:**
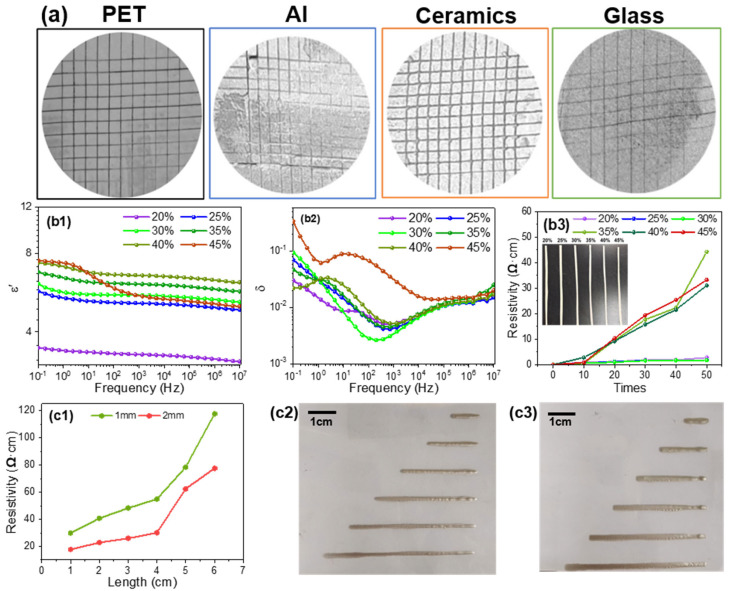
(**a**) Adhesion of silver paste film to PET, aluminum sheet, ceramic, and glass substrates; (**b1**) dielectric constants, (**b2**) dielectric loss of silver paste films with different silver contents; (**b3**) optical images and resistivity after various bending cycles of silver paste films with varying silver powder contents; (**c1**) resistivity of silver paste films with varying print widths and lengths and their optical images of screen printing at the width of (**c2**) 1 mm and (**c3**) 2 mm.

When applying conductive silver paste, consideration must be given to the preparation process, the characteristics of the substrate surface, and the adhesion between paste and substrate. The adhesion properties of silver paste are influenced by the formulation ratios of the organic solvents used as dissolving media. Taking the terpineol:DBA:DMP mass ratio of 6:3:1 as an example, such silver paste was coated on the substrates of PET, ceramic, glass, and aluminum foil. The adhesion test results are displayed in Figure 8a. Different ratios of organic solvents clearly exhibit varying degrees of adhesion to identical and dissimilar substrates (the assessment grades are shown in Table S3). Specifically, the silver paste exhibits good adhesion to PET and glass substrates, with essentially no peeling or detachment, achieving an adhesion rating of 3B or higher. However, when applied to aluminum foil and ceramic substrates, such silver paste shows significant peeling. In the case of glass substrates, the glass powder in the silver paste softens and flows at sintering temperatures, forming a strong chemical bond with the glass surface. At the same time, some of the glass phase penetrates into the micro-pores or surface defects of the substrate, creating an ‘anchoring effect’ that significantly enhances adhesion. Although PET substrates are polymers, their surfaces contain polar functional groups (such as ester and hydroxyl groups) that can form hydrogen bonds or van der Waals forces with thickeners. At the same time, the organic carrier in the silver paste partially wets the PET surface during sintering, creating mechanical interlocking and physical adsorption, resulting in good adhesion. Aluminum sheets, however, exhibit poor wettability with the glass powder in the silver paste. During sintering, the glass powder struggles to form strong chemical bonds with Al_2_O_3_. At high temperatures, the oxide layer on the surface of aluminum sheets may undergo localized reactions or stress changes, resulting in weak interfacial bonding. Although ceramics themselves are heat-resistant and have good chemical affinity with glass powder, the surface of ceramic sheets is typically dense and smooth, with low surface energy (particularly on polished surfaces). Consequently, the glass powder in the silver paste cannot form sufficient mechanical interlocking after sintering and relies solely on limited chemical adsorption, resulting in inadequate adhesion. Therefore, selecting an appropriate organic solvent for the specific substrate and carefully controlling its formulation are key to ensuring strong adhesion between the conductive silver paste and the substrate [44].

Silver paste is a commonly used conductive material in electronic devices and circuits, where electrical conductivity is paramount. However, in practical applications, it is frequently subjected to complex electromagnetic environments where factors such as frequency, temperature, and dimensions can significantly impact its performance. Consequently, conducting broadband dielectric impedance testing on conductive silver paste provides a more comprehensive understanding of its electrical capabilities and response characteristics, thereby enhancing its operational performance and stability. Figure 8(b1,b2) illustrates the dielectric properties of silver pastes prepared with varying silver content. The dielectric constant exhibits an incremental rise with an increase in silver powder content (Figure 8(b1)), followed by a subsequent decrease. Additionally, the dielectric constant exhibits an increase in its frequency-dependent behavior. This phenomenon may be attributed to the fact that, in a fixed composition paste, the interactions and aggregation of silver particles at high concentrations have the potential to influence the dielectric properties of the silver paste. As illustrated in Figure 8(b2), the dielectric loss versus frequency curve demonstrates a trough where the sample displays minimal dielectric loss. As the silver content increases, the trough shifts towards higher frequencies, concurrently with an increase in dielectric loss. This phenomenon is likely to result from the increased silver content, which enhances conductivity, thereby accelerating electron transport and consequently elevating dielectric loss [45].

Excellent resistance to bending is required for certain applications of conductive silver paste, such as membrane switches. Therefore, the selected paste must be highly flexible. From Figure 8(b3), the resistivity of silver paste film increases sharply when the silver content exceeds 30 wt.%, particularly after more than 20 bending cycles. Conversely, silver paste films with a silver content below 30 wt.% demonstrate stable resistivity, with little to no variation, even after 50 bending cycles. Furthermore, fractures and delamination occur when measuring the film with 45 wt.% silver content. This may be due to the presence of residual organic solvents during low-temperature curing, coupled with unbelted glass powder, both of which lead to the failure in formation of an optimal overlapping arrangement of silver particles [46]. Consequently, silver paste films with a silver content below 30 wt.% demonstrate superior flexural resistance.

Based on the earlier experimental studies, screen printing was conducted using a sample comprising 30 wt.% silver content and 18 wt.% glass powder to investigate the effects of line width and length during the process. The results of the resistivity test are presented in Figure 8(c1). Figure 8(c2) displays cured samples with uniform widths of 1 mm and lengths of 1 cm, 2 cm, and 3 cm, while Figure 8(c3) shows lines with a fixed width of 2 mm, with all other conditions remaining unchanged. When the screen-printing width is fixed, the resistivity undergoes an incremental increase with an increase in line length. Conversely, when the line length is fixed, increasing the screen-printing width has been shown to reduce resistivity. Furthermore, within the range of line lengths from 1 to 4 cm, resistivity exhibits a linear correlation. Following a rigorous calculation process, two linear fits are obtained, thus demonstrating high correlation coefficients of 0.987 and 0.992, respectively.

## 4. Conclusions

In this study, starting from the constituent elements of conductive silver paste, the interactions among the conductive filler, binder, thickener, and organic carrier, as well as their effects on the rheological, electrical, and mechanical properties, were systematically investigated. Through optimization of formulation composition and process parameters, effective control over the processability of the paste and the overall performance of the resulting films was achieved. In order to achieve low electrical resistivity, optimum flexural strength, and good adhesion, we selected the optimal approach as the final experimental method following appropriate experimental conditions and treatments. Conductive silver paste was prepared via a ball milling method by dispersing silver powder, glass powder, and EC in an organic carrier composed of terpineol, DBA, and DMP. Based on comprehensive evaluations of rheological behavior, electrical conductivity, bending resistance, and adhesion, the optimal formulation and process parameters were determined as follows: an organic carrier ratio of 6:3:1 (terpineol:DBA:DMP), silver powder content of 30 wt.%, glass powder content of 18 wt.%, EC content of 4 wt.%, sintering temperature of 500 °C, sintering time of 50–60 min, ball milling speed of 500–600 r/min, and ball milling time of approximately 1.5 h. Under these conditions, the silver paste exhibited pronounced shear-thinning behavior and excellent thixotropy, ensuring good screen printability. The corresponding silver paste film achieved the lowest resistivity, optimal bending resistance, and favorable adhesion to both PET and glass substrates. This work lays a solid foundation for the formulation design and process regulation of high-performance conductive silver pastes, offering valuable insights for their application in electronic packaging, flexible electronics, and automotive electronics.

## Data Availability

The original contributions presented in this study are included in the article/Supplementary Materials. Further inquiries can be directed to the corresponding author.

## Supplementary Materials

The following supporting information can be downloaded at: https://www.mdpi.com/article/10.3390/nano16100606/s1.

## References

[1] Wu J.-X., Chu C.-P., Liao Y.-C. (2023). Solderable conductive paste for electronic textiles. J. Taiwan Inst. Chem. Eng..

[2] Zareei A., Gopalakrishnan S., Mutlu Z., He Z., Peana S., Wang H., Rahimi R. (2021). Highly Conductive Copper–Silver Bimodal Paste for Low-Cost Printed Electronics. ACS Appl. Electron. Mater..

[3] Wu L., Qian J., Zhang F., Yu J., Wang Z., Guo H., Chen X. (2022). Low-Temperature Sintering of Cu/Functionalized Multiwalled Carbon Nanotubes Composite Paste for Power Electronic Packaging. IEEE Trans. Power Electron..

[4] Li C.-C., Chen C.-A., Chen M.-F. (2017). Gelation mechanism of organic additives with LiFePO4 in the water-based cathode slurries. Ceram. Int..

[5] Wang Z., Wang D., Zhang C., Chen W., Meng Q., Yuan H., Yang S. (2023). A Fluorinated Polyimide Based Nano Silver Paste with High Thermal Resistance and Outstanding Thixotropic Performance. Polymers.

[6] Ashraf M., Irshad F., Umar J., Farooq A., Ashraf M.A. (2016). Development of a novel curing system for low temperature curing of resins with the aid of nanotechnology and ultraviolet radiation. RSC Adv..

[7] Wang C., Li G., Xu L., Li J., Zhang D., Zhao T., Sun R., Zhu P. (2021). Low Temperature Sintered Silver Nanoflake Paste for Power Device Packaging and Its Anisotropic Sintering Mechanism. ACS Appl. Electron. Mater..

[8] Liu W., Wang C., Wang C., Jiang X., Huang X. (2017). Laser Sintering of Nano-Ag Particle Paste for High-Temperature Electronics Assembly. IEEE Trans. Compon. Packag. Manuf. Technol..

[9] Zhang B., Lu X., Ma H., Wang D., Mei Y.H. (2024). Development of Silver Paste with High Sintering Driving Force for Reliable Packaging of Power Electronics. IEEE Trans. Compon. Packag. Manuf. Technol..

[10] He F., Shang P., Tian Y., Zhao Z., Li N., Xie J., He Z. (2025). Study on the sintering behavior, microstructure, and properties of RO-TiO_2_-B_2_O_3_-SiO_2_ glass-ceramics applied to silver paste LTCC. J. Alloys Compd..

[11] Zou Y., Fu R., Liu X., Liu H., Wang H. (2021). Enhanced adhesion strength of silver paste on AlN ceramic substrate via sintered nano-CuO. Ceram. Int..

[12] Li H., Zhu X., Li Z., Yang J., Lan H. (2020). Preparation of Nano Silver Paste and Applications in Transparent Electrodes via Electric-Field Driven Micro-Scale 3D Printing. Nanomaterials.

[13] Chang J., Zhai H., Hu Z., Li J. (2022). Ultra-thin metal composites for electromagnetic interference shielding. Compos. Part B Eng..

[14] Lin Z., Abbott J., Karuso P., Wong D.K.Y. (2025). Advances in electroanalytical sensing of volatile organic compounds towards field-deployable detection. TrAC-Trend Anal. Chem..

[15] Zekhnini A., Delette G., Adenot-Engelvin A.L., Isnard O. (2024). Manufacturing and performances of MnZn ferrite cores with thin walls prepared by paste material extrusion 3D printing. Addit. Manuf..

[16] Park B.-G., Jung K.-H., Jung S.-B. (2017). Fabrication of the hybrid Ag paste combined by Ag nanoparticle and micro Ag flake and its flexibility. J. Alloys Compd..

[17] Cheng Y., Zhang J., Fang C., Qiu W., Chen H., Liu H., Wei Y. (2022). Preparation of Low Volatile Organic Compounds Silver Paste Containing Ternary Conductive Fillers and Optimization of Their Performances. Molecules.

[18] Nam H.J., Hwangbo Y.H., Nam S.Y., Nam H.M. (2023). Development and Performance Evaluation of Stretchable Silver Pastes for Screen Printing on Thermoplastic Polyurethane Films. Coatings.

[19] Fernandes I.J., Aroche A.F., Schuck A., Lamberty P., Peter C.R., Hasenkamp W., Rocha T.L.A.C. (2020). Silver nanoparticle conductive inks: Synthesis, characterization, and fabrication of inkjet-printed flexible electrodes. Sci. Rep..

[20] Jung J., Jang J., Chae O.B., Yoon T., Ryu J.H., Oh S.M. (2015). Reinforcement of an electrically conductive network with ethanol as a dispersing agent in the slurry preparation step. J. Power Sources.

[21] Li J., Li X., Wang L., Mei Y.-H., Lu G.-Q. (2018). A novel multiscale silver paste for die bonding on bare copper by low-temperature pressure-free sintering in air. Mater. Des..

[22] Park S., Bang J., Kim B.S., Oh S.J., Choi J.H. (2021). Metallic fusion of nanocrystal thin films for flexible and high-performance electromagnetic interference shielding materials. Mater. Today Adv..

[23] Aysin B., Ozturk A., Park J. (2013). Silver-loaded TiO_2_ powders prepared through mechanical ball milling. Ceram. Int..

[24] Traoré N.E., Schikarski T., Körner A., Cardenas Lopez P., Hartmann L., Fritsch B., Walter J., Hutzler A., Pflug L., Peukert W. (2024). Mechanistic insights into silver-gold nanoalloy formation by two-dimensional population balance modeling. Chem. Eng. J..

[25] Liu Y., Chen C., Wang Y., Zhang Z., Liu R., Ueshima M., Ota I., Nishikawa H., Nishijima M., Nakayama K.S. (2024). Development of Ag@Si composite sinter joining with ultra-high resistance to thermal shock test for SiC power device: Experiment validation and numerical simulation. Compos. Part B Eng..

[26] Fu S.-C., Zhao M., Shan H., Li Y. (2018). Fabrication of large-area interconnects by sintering of micron Ag paste. Mater. Lett..

[27] Jiang X., Xiao R., Ma Y., Zhang M., Bai Y., Huang B. (2020). Influence of waste glass powder on the physico-mechanical properties and microstructures of fly ash-based geopolymer paste after exposure to high temperatures. Constr. Build. Mater..

[28] Che Q., Yang H., Lu L., Wang Y. (2013). A new environmental friendly silver front contact paste for crystalline silicon solar cells. J. Alloys Compd..

[29] Tsai J.-T., Lin L.-K., Lin S.-T., Stanciu L., Jun M.B.-G. (2021). The influence of Bi_2_O_3_ glass powder in the silver paste and the impact on silicon solar cell substrates. Mater. Des..

[30] Nair N.M., Khanra I., Ray D., Swaminathan P. (2021). Silver Nanowire-Based Printable Electrothermochromic Ink for Flexible Touch-Display Applications. ACS Appl. Mater. Interfaces.

[31] Li Q., Liu S., Li S., Guo W., Wu C. (2016). Preparation of micro-size flake silver powder by planetary ball mill. J. Mater. Sci. Mater. Electron..

[32] (2021). Paintsandvarnishes—Cross-Cuttes.

[33] Gao Y., Feng J., Liu F., Liu Z. (2022). Effects of Organic Vehicle on the Rheological and Screen-Printing Characteristics of Silver Paste for LTCC Thick Film Electrodes. Materials.

[34] Zhang Y., Shu G., Gao S., Wang H. (2021). Viscosity-temperature characteristics of high-concentration coal-oil slurry preheated by light solvent. Fuel Process. Technol..

[35] Faddoul R., Reverdy-Bruas N., Bourel J. (2012). Silver content effect on rheological and electrical properties of silver pastes. J. Mater. Sci. Mater. Electron..

[36] Qin J., Bai S., Zhang W., Liu Z., Wang H. (2016). Effects of organic medium on rheological properties of silver pastes for crystalline silicon solar cells. Circuit World.

[37] Zdanikowski B., Szałapak J., Kądziela A., Cigula T., Vukoje M., Lepak-Kuc S., Jakubowska M., Żołek-Tryznowska Z. (2025). Development of environmentally friendly flexible electronics based on ethyl cellulose—Silver microflakes composite. Mater. Des..

[38] Yüce C., Willenbacher N. (2017). Challenges in Rheological Characterization of Highly Concentrated Suspensions—A Case Study for Screen-printing Silver Pastes. J. Vis. Exp..

[39] Wu Z., Liang Y., Fu E., Du J., Wang P., Fan Y., Zhao Y. (2018). Effect of Ball Milling Parameters on the Refinement of Tungsten Powder. Metals.

[40] Sghuri A., Billaud Y., Signor L., Saury D., Milhet X. (2023). Experimental investigation of thermal conductivity during aging of nanoporous sintered silver. Acta Mater..

[41] Sun X., Yao S., Xing J., Zhang J., Yang Y., Li H., Tong H., Yuan X. (2020). Mechanism of silver/glass interaction in the metallization of crystalline silicon solar cells. Mater. Res. Express.

[42] Li J., Wan X., Li N., Tian S., Xu L., Liu J., Ju S. (2022). Preparation of micron-sized plate-like silver powders used in silver paste by wet-chemical reduction method. J. Mater. Sci. Mater. Electron..

[43] Zhou Y., Zhang J., Chen Y., Liu S. (2021). On the isothermal sintering behavior and transparency of glass powders. J. Non-Cryst. Solids.

[44] Zhang Y., Cen Q., Xu X., Li W., Zhao Y., Li W., Liu Q., Chen M., Guo N., Wu W. (2022). The effect of PVAc in silver ink for adhesion and conductivity of conductive pattern. J. Mater. Res. Technol..

[45] Wang X.-L., Pang L.-X., Zhou D., Fang Z., Ren S., Liu W.-G. (2023). Microwave dielectric properties of (KY)1/2MoO_4_ ceramics with ultra-low sintering temperature. Mater. Today Commun..

[46] Fang J., Fu R., Zou Y., Liu H., Liu X. (2021). Hybrid silver pastes with synergistic effect of multi-scale silver fillers and the application in flexible circuits. Mater. Res. Express.

