# Supplementary material for: High-Temperature Sintered Conductive Silver Paste with Optimized Structure and Performance: Formula Design and Process Adjustment
Source: Nanomaterials (Basel). 2026 May 15;16(10):606. doi: 10.3390/nano16100606 (PMC13210059; doi:10.3390/nano16100606)
Supplement: Supplementary file 1 [file nanomaterials-16-00606-s001.zip › nanomaterials-4252090-supplementary.pdf]

High-temperature sintered conductive silver paste with optimized structure and performance: formula design and process adjustment

Gang Liu[1], Songlin Lu[2], Pengpeng Chen[2,*]

[1] Beijing Baimtec Material Co. Ltd, Beijing 100094, China

[2] School of Chemistry & Chemical Engineering, Anhui University, Hefei 230601, China

*Corresponding author.

E-mail address: chenpp@ahu.edu.cn (P. Chen).

S1 Results and discussion

S1.1 The volatility of organic solvent

Organic carrier consists of solvents and a binder. As the main component of the organic carrier, the volatility of the solvent plays a decisive role in determining the overall volatility of the organic carrier. In this study, terpineol, diethylene glycol butyl ether acetate (DBA), and dimethyl phthalate (DMP) were selected as solvents, with boiling points of 217 °C, 246 °C, and 282 °C, respectively. Solvents with different boiling points were chosen to better regulate the evaporation rate of the organic carrier. In addition, ethyl cellulose (EC) was employed as a thickener, and glass powder served as the binder. A pre-experiment was conducted to obtain an organic carrier that remained stable at room temperature, exhibiting uniformity without precipitation.

The reagents were weighed according to the designated proportions and placed in a beaker, followed by stirring in a 55 °C water bath until the EC was completely dissolved. The samples were then placed in an oven at 65 °C, 85 °C, 105 °C, 125 °C, 145 °C, 165 °C, and 185 °C for 10 min. After heating, the samples were removed and weighed, and the volatile loss was calculated by comparing the final weight with the initial weight.

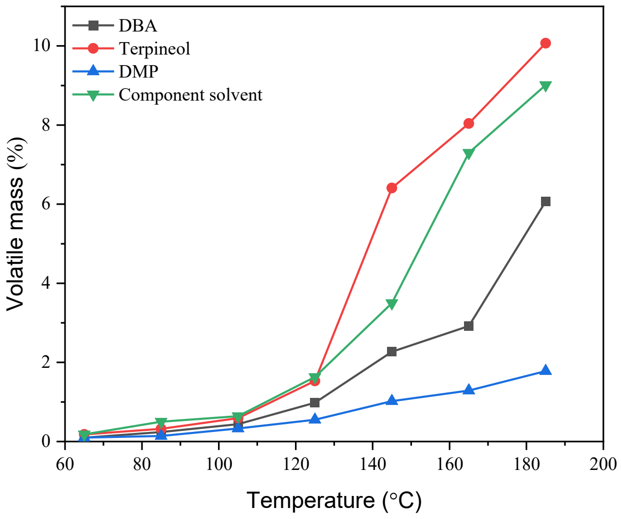

Figure S1. Volatile mass of pure solvents and component solvents

Figure S1 presents the volatility of pure solvent and component solvent. The results indicate that the volatile loss gradually increases with increasing temperature.

When the temperature exceeds 120 °C, terpineol exhibits the highest volatile loss, while DMP shows the lowest. In screen printing, excessively rapid volatilization of the organic carrier leads to a sharp increase in paste viscosity, causing screen clogging and compromising printing quality. Conversely, excessively slow volatilization results in uneven edges of the dried film, thereby deteriorating the electrical performance. Therefore, the use of a component solvent is considered the most suitable approach.

After investigating the volatility of pure and component solvents, compatibility tests between the thickener and the component solvent were further conducted. Samples were prepared according to the ratios listed in Table S1 and labeled accordingly. A mass fraction of 4% glass powder was added to each of the six sample groups, followed by ultrasonication for 30 min and subsequent standing for 30 days.

Table S1. Formulations of organic carriers with different proportions (wt.%)

Samples	①	②	③	④	⑤	⑥
EC	0.5	1	2	3	4	5
Component solvent	99.5	99	98	97	96	95

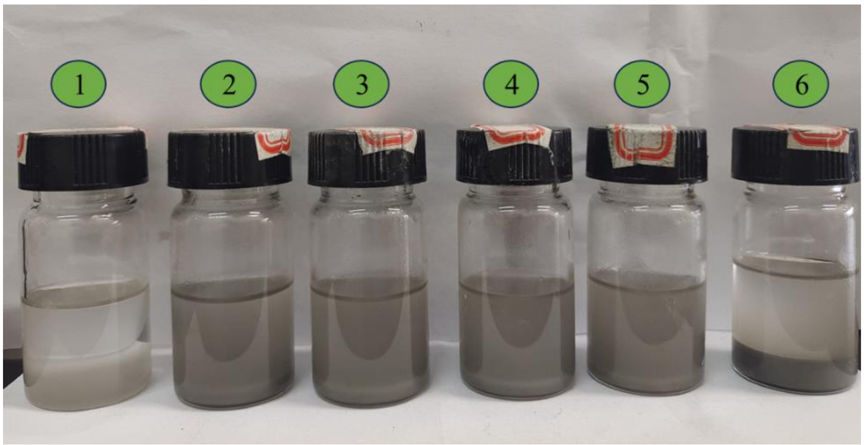

Figure S2. Compatibility of organic carriers after 30 days of standing

As shown in Figure S2, when the EC mass fraction was 0.5%, the solution remained clear and transparent; however, this EC content was too low to effectively function as a binder. When the EC content reached 5%, a substantial amount of precipitate appeared. This phenomenon is attributed to the excessive EC content, where

the organic solvent was insufficient to fully dissolve both EC and the glass powder, resulting in partially dissolved EC and glass powder encapsulating undissolved particles and settling at the bottom of the bottle. Therefore, an optimal ratio exists between EC content and the amount of organic solvent.

S1.2 The volatility of organic solvents in different proportions

The selection of terpineol (A), DBA (B) and DMP (C) as organic solvents is made, with the composition of these solvents expressed as AxByCz, where x, y and z represent the quantity of each component. Initially, the component solvents are to be weighed according to the following ratios (x = 2, 3, 4, 5, 6, 7; y = 2, 3, 4, 5, 6, 7; z = 1, 2, 3) in order to investigate the volatilisation characteristics of the mixtures at 60°C, 90°C, 120 °C, 150 °C, 180 °C, and 200 °C for 20 minutes (Table S2). The experiments were repeated a minimum of five times, and the mean experimental results were calculated to obtain the solvent volatilisation loss. It is important to note that the volatility of a given solvent is directly proportional to its boiling point, as established by Henry's Law:

$$P_B=K_{X,B}\cdot X_B \tag{S1}$$

Herein, PB denotes the pressure of the gas at the liquid surface at equilibrium, $K_{X,B}$ denotes the Henry's constant, and X_B denotes the molar fraction of solute B. Alteration of the composition ratio of the component solvent can be used to control volatility characteristics. The volatility of α-terpineol, DBA and DMP component solvents with boiling points of 217 °C-282 °C was investigated, being 217 °C, 246 °C and 282 °C, respectively.

The volatility changes of component solvents at different ratios are demonstrated in Fig. S3. The component solvents exhibit relatively low volatility between 65 °C and 205 °C, primarily due to their boiling points being significantly higher than this temperature range. As the temperature rises, the volatility of organic solvents increases concomitantly. As demonstrated in Fig. S3, formulations 5, 10, 14, and 15 demonstrate the highest rates of volatilisation at 205 °C. The low volatility, which is observed to be below 100 °C, facilitates stable slurry storage. Conversely, the high volatility, which is observed to be between 100 °C and 200 °C, promotes rapid solvent evaporation following screen printing. Following comprehensive consideration of all pertinent

factors, it is concluded that a ratio of α-terpineol, DBA and DMP of 6:3:1 in the component solvent is commensurate with the stipulated requirements.

Table S2. Ratios of different solvents

Number	x: y: z	Number	x: y: z	Number	x: y: z
1	2:7:1	6	7:2:1	11	6:2:2
2	3:6:1	7	2:6:2	12	2:5:3
3	4:5:1	8	3:5:2	13	3:4:3
4	5:4:1	9	4:4:2	14	4:3:3
5	6:3:1	10	5:3:2	15	5:2:3

Table S3. Test grade evaluation of the adhesion test of the blade

Level	Demolding Condition of Grid Scratch Area
5B	The edges of the scratch area are smooth, and there is no flaking within the grid.
4B	Minor flake-like demolding occurs in the grid area, with the demolding area less than 5%.
3B	A small amount of coating peels off along the edges and intersections of the scratches, with a peeling area of 5-15%.
2B	The coating film peels off from the edges and grid sections, with a peeling area of 15-35%.
1B	The coating peeled off in large patches across the entire scratch and grid area, with a peeling area of 35-65%.
0B	Demolding area exceeds Class 1B

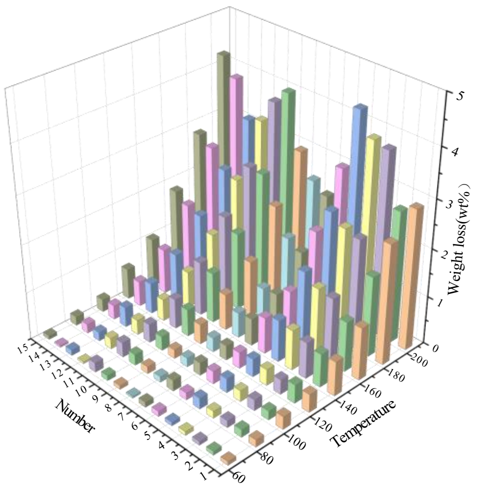

Figure S3. Volatility diagram of organic solvents of different proportions.

S1.3 Thermal properties of silver paste films

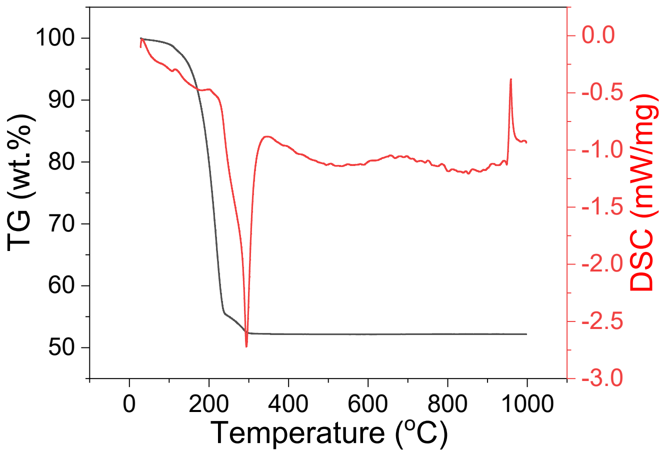

Figure S4. DSC-TG curve of silver paste.

S1.4 Electrical properties of silver paste films

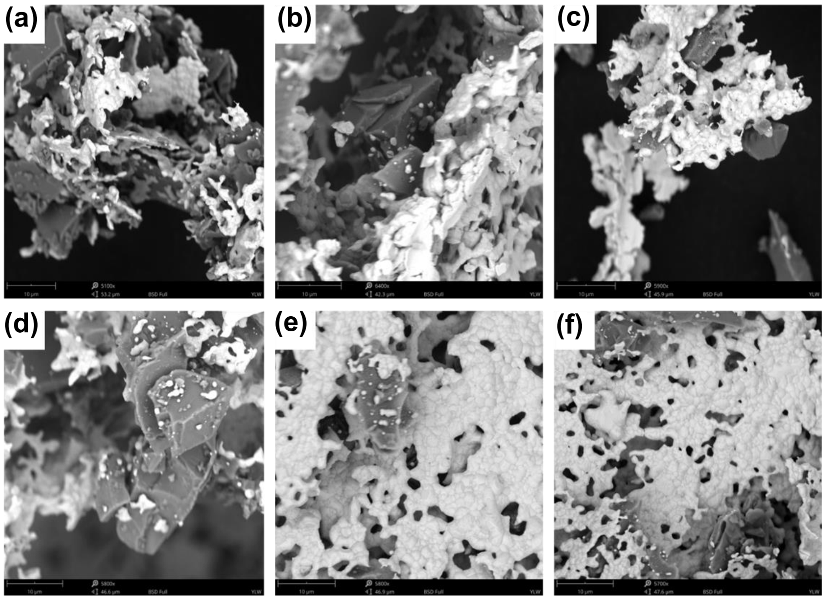

Figure S5. SEM images of silver paste films containing (**a**) 20 wt.%, (**b**) 25 wt.%, (**c**) 30 wt.%, (**d**) 35 wt.%, (**e**) 40 wt.%, (**f**) 45 wt.% silver powders.

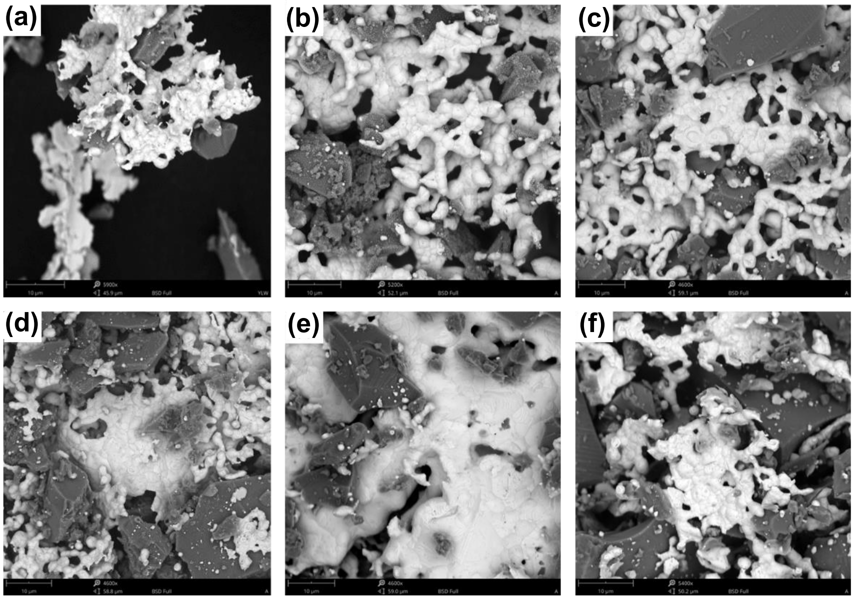

Figure S6. SEM images of silver paste films containing (**a**) 7 wt.%, (**b**) 10 wt.%, (**c**) 13 wt.%, (**d**) 15 wt.%, (**e**) 18 wt.%, (**f**) 20 wt.% glass powders.
